# Expression and clinicopathological significance of FSIP1 in breast cancer

**DOI:** 10.18632/oncotarget.3381

**Published:** 2015-03-25

**Authors:** Hao Zhang, Minna Luo, Zining Jin, Dan Wang, Ming Sun, Xinhan Zhao, Zuowei Zhao, Haixin Lei, Man Li, Caigang Liu

**Affiliations:** ^1^ Breast Disease and Reconstruction Center, Breast Cancer Key Lab of Dalian, The Second Hospital of Dalian Medical University, Dalian, China; ^2^ Department of Oncology, The First Affiliated Hospital, Xi'an Jiaotong University, Xi'an, China; ^3^ Department of Breast surgery, The First Hospital of China Medical University, Shenyang, China; ^4^ Shengjing Hospital, China Medical University, Shenyang, China; ^5^ Institute of Cancer Stem Cell, Cancer Center, Dalian Medical University, Dalian, China

**Keywords:** breast cancer, prognosis, metastasis, FSIP1

## Abstract

**Aim:**

To investigate the clinicopathological significance of the expression of fibrous sheath interacting protein 1 (FSIP1) in breast cancer, serum samples, and wound fluid from patients with breast cancer.

**Methods:**

Wound fluid and serum samples from female patients with primary breast cancer, recurrent and metastatic breast cancer, and benign tumors were analyzed for FSIP1 expression using ELISA. 286 paraffin-embedded surgical specimens from breast cancer patients with at least 5 years of follow-up were included for FSIP1 expression assay using immunohistochemistry.

**Results:**

Expression of FSIP1 protein was significantly higher in breast cancer tissues compared to tumor-adjacent tissues (*p* = 0.001). Strong correlation was observed between FSIP1 expression and human epidermal growth factor receptor 2 (Her-2) or Ki67 expression in breast cancer (*p* = 0.027 and 0.002, respectively). Similarly, serum level of FSIP1 was higher in patients with recurrent and metastatic breast cancer compared to that of primary breast cancer (7, 713 ± 3, 065 vs. 4, 713 ± 3, 065 pg/ml, *p* = 0.003). Finally, patients with high FSIP1 expression showed a worse post-operative disease-specific survival (*p* = 0.024).

**Conclusion:**

FSIP1 may play an important role in the tumorigenesis and invasion of breast cancer and is a potential biomarker for breast cancer diagnosis or prognosis.

## BACKGROUND

Currently, the primary treatment for breast cancer is surgery followed by chemotherapy, radiotherapy or endocrine therapy, while targeted treatments are employed to eliminate residual tumor cells and thus reduce the risk of recurrence and metastasis [1–3]. Some patients, however, still show relapse or metastasis after post-operative therapy. The reason that post-operative therapies failed or not all patients responded to targeted therapy remains elusive. Therefore, it is urgent to identify novel biomarkers that can discriminate these refractory patients. More importantly, it is crucial to discover new therapeutic targets with high specificity via further understanding on the molecular mechanisms underlying tumorigenesis and metastasis of breast cancer [4, 5].

*FSIP1* is a recently discovered gene that encodes fibrous sheath interacting protein 1 (FSIP1). Expression of *FSIP1* is known to be regulated by amyloid beta precursor protein [6]. FSIP1 is a potential target for cancer therapy since its mRNA level is undetectable in most normal tissues and its expression is elevated in breast tumors. However, the previous study only included a small sample size and did not correlate FSIP1 expression level with prognosis [7]. Therefore, further study with large sample size is required to clarify the role of FSIP1 in breast cancer.

The aim of this study was to investigate the protein expression of FSIP1 in breast cancer, and to build up the correlation between FSIP1 expression and the clinicopathological features and prognosis of breast cancer.

## RESULTS

### FSIP1 expression in breast cancer and its correlation with clinicopathological characteristics

**Figure 1:**
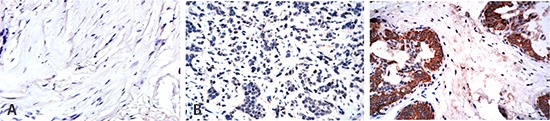
Higher expression of FSIP1 in Her-2 positive cases FSIP1 expression in 286 surgical tissue samples from patients with breast cancer was detected by performing immunohistochemistry. (A) Negative FSIP1 staining on the myoepithelial cells in the surrounding non-tumor areas; (B) Negative FSIP1 staining in ER-positive breast cancer; (C) Positive FSIP1 staining in Her-2 positive breast cancer.

In total, 45.45% of the cases showed high FSIP1 expression in breast tumor tissue with no expression in tumor-adjacent tissues (*p* = 0.001; Figure 1 and Table 1). FSIP1 protein was expressed at higher levels in human epidermal growth factor receptor 2 (Her-2) positive breast cancer tissues compared to Her-2 negative tissues (*p* = 0.029). Similarly, FSIP1 expression level was considerably higher in samples with more than 14% Ki67 expression compared to those with less than 14% (*p* = 0.002). No correlation between FSIP1 expression and age, tumor size, estrogen receptor (ER) status, or progesterone receptor (PR) status was observed (Table 1). Spearman correlation analysis revealed strong correlations between lymph node metastasis, Her-2 and Ki67 expression status and FSIP1 expression (*p* = 0.009, 0.027 and 0.002, respectively; Table 2).

**Table 1:** Correlations between FSIP1 expression and clinicopathological features (*n* = 286)

Variables	*N*	FSIP1^−^	FSIP1^+^	*p* value
**Age**				0.768
≤ 45 Y	75	42	33	
> 45 Y	211	114	97	
**Tumor size**				0.953
Tis	8	5	3	
T1	118	63	55	
T2	152	84	68	
T3	8	4	4	
**ER status**				**0.451**
negative	86	44	42	
positive	200	112	88	
**PR status**				0.873
negative	96	53	43	
positive	190	103	87	
**Her-2 status**				**0.029**
negative	262	148	114	
positive	24	8	16	
**Ki-67 status**				**0.002**
≤ 14%	220	131	89	
>14%	66	25	41	

The *p* value was calculated using chi-square test or fisher's extract test.

**Table 2:** Correlation analysis between clinicopathological features and FSIP1 expression

Clinicopathological features	FSIP1 expression (*p* value; Spearman correlation)
Age	0.905 (0.007)
Tumor size	0.837 (0.012)
Lymph node metastasis	0.009 (0.155)
ER	0.439 (0.047)
PR	0.896 (0.008)
Her-2 status	0.027 (0.132)
Ki67	0.049 (0.128)

Note: 8 DCIS cases excluded.

### Serum level of FSIP1 in primary breast cancer patients

**Figure 2:**
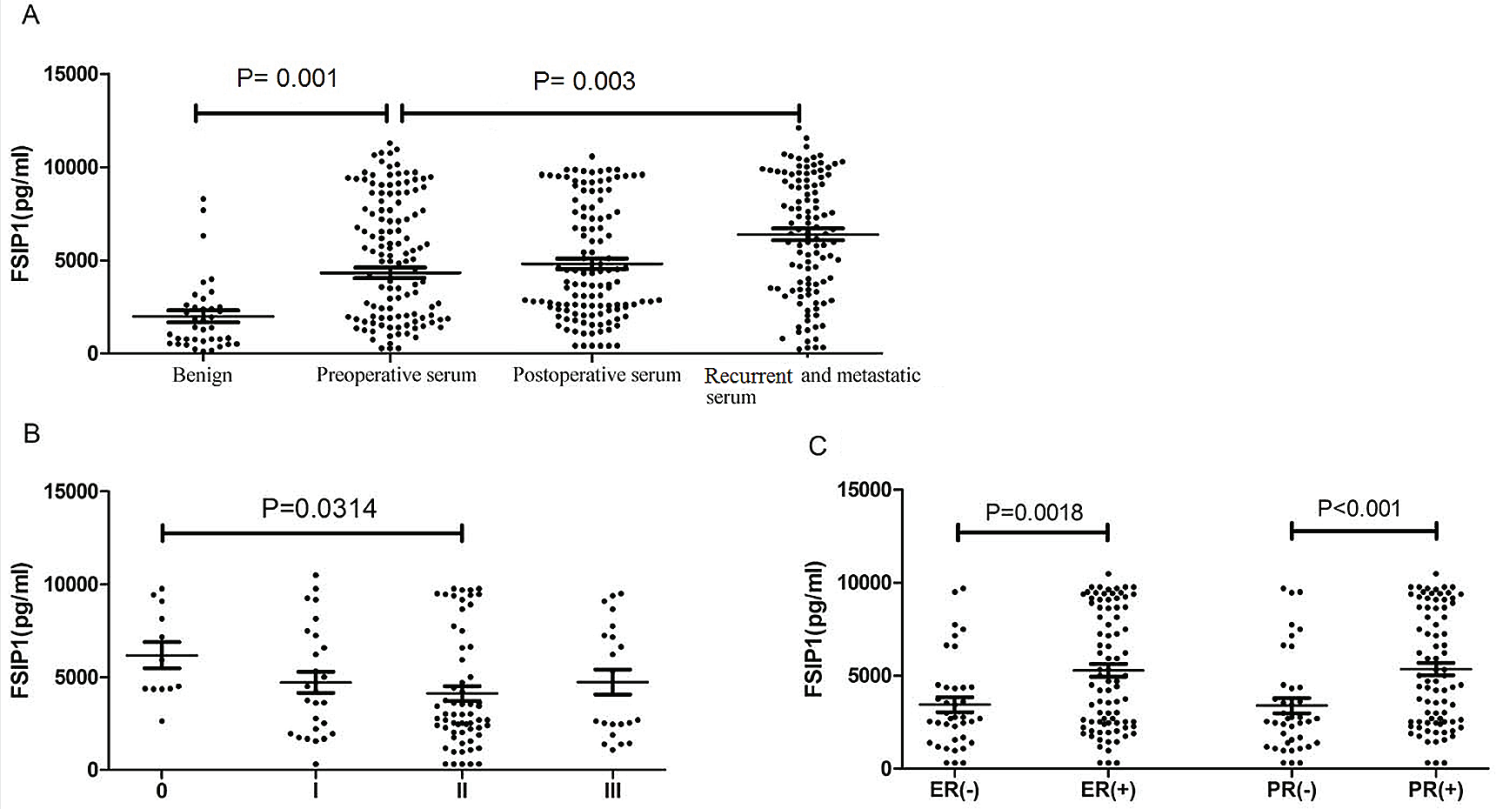
Serum FSIP1 expression levels in breast cancers (A) There was no significant difference in serum FSIP1 levels before and after breast cancer surgery in 122 patients (*p* = 0.162). Serum FSIP1 levels in patients with primary breast cancer were significantly higher than that in patients with benign disease (*p* < 0.0001). Serum FSIP1 levels in 112 patients with recurrent and metastatic breast cancer were significantly higher than those of patients with primary breast cancer (*p* = 0.003). (B) The level of FSIP1 was correlated to clinical stages of breast cancer. Serum levels of FSIP1 between stage 0 and stage II cancers were significantly different (stage 0 vs. stage II: *p* = 0.0314). (C) FSIP1 levels in breast cancer patients with ER- or PR-positive expression were significantly higher than those with ER- or PR-negative expression (*p* = 0.0018 and *p* < 0.0001, respectively).

There was no significant difference in serum FSIP1 levels before and after surgery in the 122 patients with primary breast cancer (4, 637 ± 3, 276 pg/ml vs. 4, 713 ± 3, 065 pg/ml, *p* = 0.162; Figure 2). However, serum FSIP1 level was significantly higher in patients with primary breast cancer than in patients with benign cancer (4, 713 ± 3, 065 pg/ml vs. 1, 798 ± 1, 943 pg/ml, *p* = 0.001). Serum FSIP1 level in the 112 patients with recurrent or metastatic breast cancer was also much higher than those of the patients with primary breast cancer (7, 713 ± 3, 065 pg/ml vs. 4, 713 ± 3, 065 pg/ml, *p* = 0.003; Figure 2A).

No difference was found in serum FSIP1 level between the ductal carcinoma *in situ* group and the invasive breast cancer group (6, 172 ± 2, 432 pg/ml vs. 4, 381 ± 3, 019 pg/ml, *p* = 0.3493, Table 3). Serum FSIP1 concentration was 4, 785 ± 2, 843 pg/ml, 4, 230 ± 3, 174 pg/ml, and 2, 476 ± 1, 997 pg/ml in T1, T2 and T3 tumors, respectively (*p* = 0.2148, Table 3). In addition, serum FSIP1 expression level in stage 0, I, II and III tumors was 6, 172 ± 2, 432 pg/ml, 4, 720 ± 2, 916 pg/ml, 4, 114 ± 3, 065 pg/ml, and 4, 734 ± 3, 075 pg/ml, respectively (*p* = 0.2314; Figure 2B).

**Table 3:** Clinicopathological implications of FSIP1 levels in breast cancer serum

Variables	*N*	FSIP1 (pg/ml) (Mean ± SD)	*p*-value
**Age**			0.5983
≤ 45 years old	59	4, 347 ± 2, 884	
> 45 years old	63	4, 629 ± 2, 999	
**Histological type**			0.3493
IBC	110	4, 381 ± 3, 019	
DCIS	12	6, 172 ± 2, 432	
**Histological grade**			0.2314
0	12	6, 174 ± 2, 432	
I	27	4, 720 ± 2, 916	
II	62	4, 114 ± 3, 065	
III	21	4, 734 ± 3, 075	
**Tumor size**			0.0148*
Tis	12	5, 919 ± 2, 536	
T1(≤ 2)	39	4, 785 ± 2, 843	
T2 (> 2, ≤ 5)	69	4, 230 ± 3, 174	
T3 (> 5)	4	2, 476 ± 1, 997	
**Metastatic nodes**			0.0401*
Positive	43	3, 943 ± 2, 630	
Negative	79	5, 132 ± 3, 216	
**ER status**			0.0018*
Positive	84	5, 286 ± 3, 152	
Negative	38	3, 445 ± 2, 458	
**PR status**			< 0.001*
Positive	82	5, 357 ± 3, 066	
Negative	40	3, 392 ± 2, 638	
Her-2 status			0.3568
Positive	20	4, 133 ± 2, 119	
Negative	102	4, 827 ± 3, 214	
**Ki-67 status**			0.6154
> 14%	102	4, 651 ± 3, 119	
≤ 14%	20	5, 029 ± 2, 824	
**Molecular subtypes**			0.2168
Luminal A	16	4, 749 ± 2, 917	
Luminal B	70	5, 383 ± 3, 170	
Her-2 over expression	14	2, 917 ± 2, 241	
Triple negative	22	3, 697 ± 2, 683	

Abbreviation: N: number, IBC: invasive breast cancer, T; tumor size, Tis: tumor in situ, DCIS: ductal carcinoma in situ, ER: estrogen receptor, PR: progesterone receptor, Her-2: human epithelial receptor-2.

* *p* < 0.05 (significant association)

Serum FSIP1 level in patients with ER-positive breast cancer was significantly higher than that in ER-negative cases (5, 286 ± 3, 152 pg/ml vs. 3, 445 ± 2, 458 pg/ml, *p* = 0.0018). Similarly, serum level of FSIP1 in patients with PR-positive breast cancer was significantly higher than that in PR-negative cases (5, 357 ± 3, 066 pg/ml vs. 3, 392 ± 2, 638 pg/ml, *p* < 0.0001; Figure 2C). However, no difference in serum FSIP1 level was observed in patients with different molecular types of breast cancer (*p* = 0.2168, Table 3).

**Figure 3:**
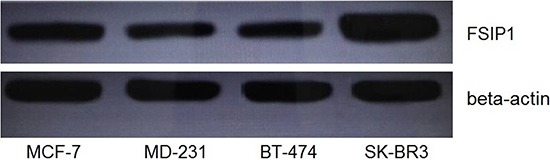
FSIP1 protein expression in breast cancer cell lines Western blot analysis showed that intracellular FSIP1 levels were significantly higher in SK-BR3 (Her-2 positive) than in MCF-7 (luminal A positive), BT-474 (luminal B positive), or MD-231 (triple negative) cell lines.

We also checked the expression level of FSIP1 in four typical breast cancer cell lines, including MCF-7 (luminal A), BT-474 (luminal B), MD-231 (triple negative), and SK-BR3 (Her-2 over expression). Intracellular FSIP1 protein level was apparently higher in the SK-BR3 cell line than in the other three cell lines (Figure 3), which was consistent with the observation on immunohistochemistry (Figure 1 and Table 1) and Spearman correlation regression analysis (Table 2).

### Level of FSIP1 in wound fluid of breast cancer patients after surgery

**Figure 4:**
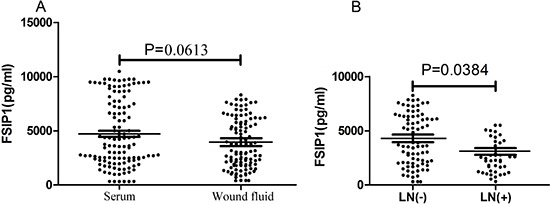
FSIP1 expression level in wound fluid after surgery (A) There was no difference in FSIP1 level between wound fluid and serum of patients with primary breast cancer (*p* = 0.0613). (B) FSIP1 level in wound fluid of patients with negative lymph nodes was higher than those of patients with positive lymph nodes (*p* = 0.0384).

No difference in the levels of FSIP1 between the wound fluid and serum sample of patients with primary breast cancer was observed (4, 613 ± 3, 612 pg/ml vs. 4, 713 ± 3, 065 pg/ml, *p* = 0.0613) (Figure 4A). However, FSIP1 expression in the wound fluid from patients with negative lymph nodes was significantly higher than in wound fluid from patients with positive lymph nodes (4, 937 ± 2, 914 pg/ml vs. 3, 273 ± 2, 647 pg/ml, *p* = 0.0384; Figure 4B).

### Prognosis analysis

**Figure 5:**
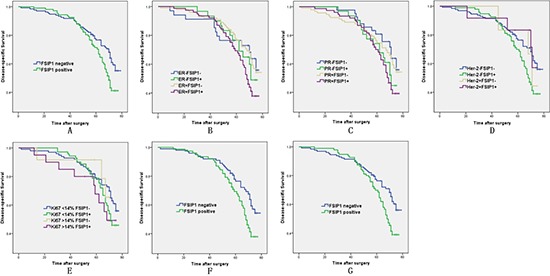
FSIP1 expression is associated with poor survival of breast cancer patients Association between survival of 278 patients with invasive breast cancer and FSIP1 expression was estimated using Kaplan-Meier method and analyzed using log-rank test. (A) Patients with high FSIP1 expression had a worse postoperative disease-specific survival compared to the ones with negative FSIP1 expression (*p* = 0.022). Cumulative survival curves of FSIP1-positive and FSIP1-negative cancers according to ER, PR, Her-2, and Ki67 statuses are shown in Figure 5B–5E. Significant survival differences were observed between FSIP1-positive status and FSIP1-negative status in patients with ER-positive and Her-2-negative tumors (*p* = 0.016 and 0.009, respectively; Figures 5F and 5G).

Patients with high FSIP1 expression in tumors tended to have worse post-operative disease-specific survival (*p* = 0.022; Figure 5A). When the data were analyzed according to the expression status of ER, PR, Her-2, and Ki67 in each tumor (Figure 5B–5E), significant survival differences were observed between FSIP1-positive status and FSIP1-negative status in patients with ER-positive and Her-2 negative tumors (*p* = 0.016 and 0.009, respectively; Figures 5F and 5G). The hazard ratio for death was 1.578 (95% CI, 1.062–2.345; *p* = 0.024) in the FSIP1-positive group (univariate analysis). After adjustment of seven baseline variables (age, tumor size, lymph node status, ER status, PR status, Her-2 status, and Ki67 status) by using Cox regression analysis, the hazard ratio was not significantly changed (hazard ratio, 1.383 [95% CI, 0.871–2.195]; *p* = 0.081; Table 4).

**Table 4:** Hazard ratio for disease free survival

	Univariable analysis		Multivariable analysis	
	HR (95% CI)	p*	HR (95% CI)	*p*^†^
**Age (years)**		**0.180**		**0.983**
≤ 45	1 (Ref)		1 (Ref)	
> 45	1.371 (0.865–2.176)		1.042 (0.626–1.735)	
**Tumour stage**		**0.166**		**0.295**
T1	1 (Ref)		1 (Ref)	
T2	1.126 (0.749–1.694)	0.567	1.183 (0.745–1.878)	0.475
T3	2.981 (1.170–7.597)	0.022	2.358 (0.686–8.099)	0.173
**Lymph-node stage**		**0.906**		**0.501**
N0	1 (Ref)		1 (Ref)	
N1	0.836 (0.483–1.447)	0.523	0.612 (0.307–1.220)	0.163
N2	1.606 (0.914–2.820)	0.100	1.318 (0.677–2.565)	0.417
N3	0.631 (0.230–1.734)	0.372	0.522 (0.159–1.715)	0.284
**ER status**		**0.462**		**0.460**
Negative	1 (Ref)		1 (Ref)	
Positive	1.186 (0.753–1.868)		1.208 (0.625–2.335)	
**PR status**		**0.353**		**0.453**
Negative	1 (Ref)		1 (Ref)	
Positive	1.229 (0.796–1.897)		1.140 (0.631–2.059)	
**Her-2 status**		**0.509**		**0.575**
Negative	1 (Ref)		1 (Ref)	
Positive	0.772 (0.358–1.666)		0.760 (0.322–1.795)	
**Ki-67 status**		**0.568**		**0.567**
≤ 14%	1 (Ref)		1 (Ref)	
> 14%	1.198 (0.645–2.223)		1.152 (0.602–2.204)	
**FSIP1 status**		**0.024**		**0.081**
Negative	1 (Ref)		1 (Ref)	
Positive	1.578 (1.062–2.345)		1.383 (0.871–2.195)	

Note: *n* = 278, with 8 DCIS cases excluded.

Ref: reference category.

* Derived from tests of HR for prognostic factors in univariate model adjusted for treatment group in Cox proportional-hazards model.

† Cox-regression analysis, controlling for prognostic factors listed in table.

## DISCUSSION

FSIP1 is a component of fibrous sheath in sperm flagellum that assembles AKAP4, which was the original X-linked CT antigen detected in breast cancer [9]. It is well-known that protein kinase A (PKA) plays an important role in tumor proliferation, angiogenesis, and chemoresistance [10–13]. AKAP4 has been reported to be one of the scaffolding proteins associated with cAMP-dependent PKA [10]. Multiple studies have shown that AKAP4 is strongly expressed in several types of cancer [9, 14]. As a component of AKAP4, FSIP1 may play a role in tumorigenesis and could therefore be a target for cancer immunotherapy. It has also been shown that FSIP1 functions in the regulation of chromosome segregation in tumor cells [15]. In addition, there is evidence to support that FSIP1 is a target of steroid receptor coactivator-3 [16], which is an oncogene associated with breast cancer [17] and a coactivator for nuclear receptors, such as ER-α [18]. However, the expression levels and clinical implications of FSIP1 expression in breast cancer and especially in the serum and wound fluid were still unclear.

In the present study, a cohort of 286 breast cancer samples was assayed for FSIP1 expression. The results indicated that FSIP1expression was significantly higher in breast cancer tissues compared to benign tissues, and FSIP1 expression in breast cancer was found to be correlated with a worse post-operative disease-specific survival. Moreover, FSIP1 expression was significantly correlated to Her-2 and Ki-67 expression but not to ER or PR level. However, Chapman *et al* reported higher FSIP1 expression in ER-positive breast tumors compared to ER-negative breast tumors [7], such conflict may due to different methods for FSIP1 quantification, number of samples verified with FSIP1 protein expression or different ethnic origins. We tested the level of FSIP1 protein in breast cancer cell lines in order to confirm the outcomes of the clinical data. The Her-2 positive cell line SK-BR3 expressed higher level of FSIP1 compared to the other cell lines including MCF-7 (an ER positive cell line). We are now studying the function of FSIP1 in Her-2 positive breast cancers.

We also quantified FSIP1 expression in serum and wound fluid to determine whether FSIP1 could be secreted into the wound or blood, which may have an impact on the dissemination of residual tumor cells after surgery. We observed that FSIP1 was highly expressed in the serum of recurrent and metastatic breast cancer compared to primary breast cancer. Furthermore, FSIP1 expression level in the tumor significantly predicted distant metastasis in prognosis analysis. Further functional study is needed to address how FSIP1 might regulate tumor metastasis.

## MATERIALS AND METHODS

### Patients and samples

Blood serum samples from 122 female patients with primary breast cancer (mean age: 52.5 ± 8.3 years), 112 patients with recurrent and metastatic breast cancer (mean age: 56.3 ± 11.6 years) and 38 patients with benign tumor (mean age: 37.2 ± 10.8 years) were included in this study. Serum samples were collected within 1 week before surgery and 3 days after surgery, wound fluid was collected 2 and 3 days after surgery. For the 122 patients with primary breast cancer, no mastectomy, breast-conserving surgery or systemic treatment for breast cancer was performed before undergoing primary breast cancer surgery at the 2^nd^ affiliated hospital of Dalian Medical University between 2011 and 2013. In addition, 286 paraffin-embedded breast cancer tissues from patients with at least a 5-year follow-up were assayed for FSIP1 protein expression using immunohistochemistry and included in prognosis analysis.

The diagnosis of all patients met the criteria of modified National Comprehensive Cancer Network Clinical Practice Guidelines in Oncology-Breast Cancer Guideline 2012. The present study was approved by the ethics committee of the 2^nd^ affiliated hospital of Dalian Medical University, according to the Declaration of Helsinki. All individuals provided written consent for participation in the study.

### Assay for FSIP1 level in wound fluid and serum

FSIP1 level in wound fluid and serum was measured using an enzyme-linked immunosorbent assay (ELISA) in accordance with the manufacturer's recommendation (R&D Systems, USA).

### Immunohistochemistry

Procedure was the same as previously described with minor modifications [8]. Briefly, 4-μm breast tumor tissues were cut using a cryostat. Sections were mounted on microscope slides, fixed in a mixture of 50% acetone and 50% methanol after air dry. Samples were then de-waxed in xylene, gradually hydrated with gradient alcohol, and washed with phosphate buffered saline (PBS). After that, sections were incubated for 60 min with rabbit polyclonal FSIP1 antibody (1:500 dilution, Santa Cruz Biotechnology, USA). Following PBS wash, sections were further incubated for 30 min with the secondary biotinylated antibody (Multilink swine anti-goat/mouse/rabbit immunoglobulin; Dako Inc., Denmark). Next, an avidin biotin complex (1:1000 dilution, Vector Laboratories Ltd., United Kingdom) was applied to the sections and incubated for 30–60 min at room temperature. The immunoreactive products were visualized by catalysis of 3, 3-diaminobenzidine with horseradish peroxidase (HRP) in the presence of H_2_O_2_. Last, sections were counterstained with Gill's hematoxylin and dehydrated in ascending grades of methanol, before clearing in xylene and mounting under a coverslip. As a negative control, staining was performed in parallel without primary antibody.

Reactivity of anti-FSIP1 antibody was showed as brown granules located at tumor cell membrane/cytoplasm and graded as follows: 0, no staining; 1, partial staining of the membrane/cytoplasm; 2, mild to moderate circumferential staining of the membrane/cytoplasm; and 3, strong circumferential staining of the membrane/cytoplasm. A score of 2 or 3 was considered positive for FSIP1 expression.

### Western blot analysis

Total proteins were extracted using a protein extraction kit (ProMab, USA) followed by centrifugation. Protein concentration was quantified using BCA assay (Santa Cruz Biotechnology, USA), individual cell lysate (30 μg/lane) was then separated on sodium dodecyl sulfate polyacrylamide gel and transferred onto polyvinylidene fluoride membranes. Membrane was blocked with 5% fat-free dry-milk in TBST and incubated with rabbit anti-FSIP1 antibody (1:100 dilution; Abcam, USA) followed by rabbit anti- β-actin antibody (1:5000 dilution; Abcam) at 4°C overnight. Bound antibodies were detected with HRP-conjugated anti-rabbit, anti-mouse, or anti-goat immunoglobulin G (IgG) at room temperature for 1 h and visualized with enhanced chemiluminescence (Santa Cruz Biotechnology, USA). Purified mouse, rabbit, or goat IgG was used as a negative control. Relative levels of targeting protein to the control β-actin were determined using ImmuNe software.

### Statistical analysis

Statistics was performed with SPSS Statistics software, version 16.0. Experimental data are presented as mean ± standard error. Continuous variables from the study were analyzed using ANOVA and/or Student *t*-test (with a parametric distribution) or Mann-Whitney U test (with a nonparametric distribution). Spearman correlation coefficient was applied to test for correlations between two variables. Survival analysis was performed using Kaplan–Meier method and comparisons were made by using log-rank test. Hazard ratios and corresponding 95% confidence intervals (CIs) were calculated using Cox proportional hazards model. Statistic significance was set at *p* < 0.05.

## CONCLUSION & FUTURE PERSPECTIVES

Our data support that FSIP1 is a potential biomarker for early diagnosis and prognosis of breast cancer, together with previous reports, FSIP1 maybe a novel target for breast cancer therapy. So far, little is known about the role of FSIP1 in cancer development, we are working on defining the mechanism by which FSIP1 contributes to tumorigenesis and metastasis.
